# Modelling innovative interventions for optimising healthy lifestyle promotion in primary health care: "*Prescribe Vida Saludable*" phase I research protocol

**DOI:** 10.1186/1472-6963-9-103

**Published:** 2009-06-18

**Authors:** Alvaro Sanchez, Gonzalo Grandes, Josep M Cortada, Haizea Pombo, Laura Balague, Carlos Calderon

**Affiliations:** 1 Primary Care Research Unit of Bizkaia, Basque Health Service (Osakidetza), Bilbao, Spain; 2 Deusto Health Centre, Basque Health Service (Osakidetza), Bilbao, Spain; 3 Renteria Health Centre, Basque Health Service (Osakidetza), Renteria, Spain; 4 Alza Health Centre, Basque Health Service (Osakidetza), Donostia-San Sebastian, Spain

## Abstract

**Background:**

The adoption of a healthy lifestyle, including physical activity, a balanced diet, a moderate alcohol consumption and abstinence from smoking, are associated with large decreases in the incidence and mortality rates for the most common chronic diseases. That is why primary health care (PHC) services are trying, so far with less success than desirable, to promote healthy lifestyles among patients. The objective of this study is to design and model, under a participative collaboration framework between clinicians and researchers, interventions that are feasible and sustainable for the promotion of healthy lifestyles in PHC.

**Methods and design:**

Phase I formative research and a quasi-experimental evaluation of the modelling and planning process will be undertaken in eight primary care centres (PCCs) of the Basque Health Service – OSAKIDETZA, of which four centres will be assigned for convenience to the Intervention Group (the others being Controls). Twelve structured study, discussion and consensus sessions supported by reviews of the literature and relevant documents, will be undertaken throughout 12 months. The first four sessions, including a descriptive strategic needs assessment, will lead to the prioritisation of a health promotion aim in each centre. In the remaining eight sessions, collaborative design of intervention strategies, on the basis of a planning process and pilot trials, will be carried out. The impact of the formative process on the practice of healthy lifestyle promotion, attitude towards health promotion and other factors associated with the optimisation of preventive clinical practice will be assessed, through pre- and post-programme evaluations and comparisons of the indicators measured in professionals from the centres assigned to the Intervention or Control Groups.

**Discussion:**

There are four necessary factors for the outcome to be successful and result in important changes: (1) the commitment of professional and community partners who are involved; (2) their competence for change; (3) the active cooperation and participation of the interdisciplinary partners involved throughout the process of change; and (4) the availability of resources necessary to facilitate the change.

## Background

Although it is well known that the most common chronic diseases are caused by unhealthy behaviours such as smoking and drinking alcohol, a sedentary lifestyle and an unbalanced diet; in spite of primary health care (PHC) services having many opportunities to intervene in those lifestyles; and despite the fact that its professionals are convinced that healthy lifestyle promotion by health services has a potential impact that few other interventions could match; the truth is that healthy lifestyle promotion is far from being an integrated element of clinical practice in PHC [1]. The two main reasons for this are: a) insufficient evidence of the effect of interventions and active components on multiple risk behaviours [2,3]; and b) the difficulties in changing existing practice to include innovative interventions under real-world conditions [4,5].

To date, interventions on lifestyle modification in PHC have shown mixed results [2,3]. There is solid evidence proving the effectiveness of brief advice given in PHC in achieving smoking cessation and reductions in alcohol consumption [6-8]. In the case of physical activity, general advice has little effects, while, medical guidance in the form of a written prescription for physical activity results in small or moderate changes even in the long term [9,10]. However, concerning diet and the simultaneous approach of several behavioural risk factors, there is still insufficient evidence on the effectiveness of the advice given in PHC [11-13]. Even for those lifestyles for which there is evidence of effectiveness, it is not clear which are the effective components of the interventions, and there is high heterogeneity across the results obtained depending on the context and the way in which the interventions are implemented [3].

Changing people's lifestyles is not easy, because their behaviour is determined by many personal, institutional and environmental factors, which operate and interact at individual, interpersonal and community levels [14]. Consequently, there is a growing recognition of the need to base lifestyle change interventions on relevant behaviour change theories and to follow a suitable process linking intervention techniques and strategies with this theory. This, as well as producing more effective interventions, should make it possible to identify which factors work, how they work and why [15]. Classical planning models such as Precede-Proceed [16] or more modern ones such as Intervention Mapping and Causal Modelling [17,18], as well as consensus techniques and constructs of behaviour change [19] and guidelines such as those of NICE concerning the development of behaviour change interventions [20], could facilitate this process of mapping and evaluation of theories and effective techniques.

The improvement of preventive clinical practice involves the transferring and dissemination of effective and efficient interventions to real-world clinical conditions [21]. The failure to date in translating identified effective clinical interventions into routine practice represents the gap between what is avoidable or preventable via these interventions and what is achieved in practice. Several types of quality improvement interventions have been developed with the objective of reducing this gap. In general, what has been recommended is the use of multifaceted interventions that include several strategies such audits and feedback, external facilitators, evidence-based educational meetings with active participation and knowledge management. Specifically, in the area of optimisation of prevention services, efficient registration and reminding systems, a revision of professionals' roles, nursing-based programmes, the creation of multidisciplinary teams, integrated care services and collaboration with community resources, have shown positive results [22-34].

However, the evidence on translation of effective preventive interventions, strategies and programmes is at present insufficient or not conclusive [35]. On the one hand, the effectiveness of the majority of these strategies is low or moderate with a high variation in the degree of change achieved. On the other hand, interventions that have had some success are not easily incorporable to the real-world context of health centres [36]. This is why it has been recommended that interventions for the optimisation of preventive clinical practice should be adapted to the real context of each centre and health system: to their needs, characteristics and identified barriers [37-39]. In line with this, recent models concerning translation of evidence into practice propose the optimisation of clinical practice through research [40-42]. Under this framework, research should be used to optimise practice instead of using the practice context to attempt to demonstrate the relevance of previous studies. Interventions should be designed in the same context in which they are going to be executed, with the active participation of the principle players [41].

There are many challenges in the design, evaluation and transfer of healthy lifestyle promotion to the clinical context, mainly due to the complexity of the interventions [43-45]. They are composed of a large number of elements and are focussed on a variety of interrelated levels: the patient as an individual, health professionals and the organisation that offers health services to the community, in a context that is characterised by work overload and lack of time. In 2000, the Medical Research Council (MRC) of the United Kingdom defined a theoretical and methodological framework for the design and evaluation of this type of complex interventions in the clinical context [43]. This framework, updated in 2008 [45], uses simultaneously qualitative and quantitative techniques, and is developed in a series of phases, similar to those of clinical drug research, that could be executed in a sequential or iterative manner: a) preclinical or theoretical phase: establishment of theoretical fundamentals and identification of the active components in the evidence base; b) phase I or modelling phase: definition of intervention components, identification of potential barriers to change and of the mechanisms through which interventions should operate; c) phase II or exploratory trial: evaluation of the feasibility and optimisation of the intervention through the execution of quasi-experimental studies; d) phase III or definitive randomised controlled trial, to enable the controlled experimental evaluation of the intervention; e) phase IV or long term implementation phase under real-world conditions. To date, there have been several projects that have successfully applied the MRC framework for the design and evaluation of complex interventions [45]. The conclusions of these studies agree on the usefulness of the MRC framework as a tool for the researchers in the designing, planning and evaluation of innovative interventions to improve health.

In 2006, in order to tackle the problem of integrating health promotion into PHC, the Primary Care Research Unit of the Basque Health Service set up a multidisciplinary team of 12 health professionals: family doctors, paediatricians and nurses in PHC, specialists in preventive medicine, public health, health education, epidemiologists, psychologists and sociologists. Between 2006 and 2007, this group undertook a first preclinical research phase on usefulness of various theoretical models and intervention strategies for health behaviour change (sedentary lifestyle, diet, smoking, alcohol), and identified factors that make their integration in PHC difficult [1]. In line with the conclusions of this work, the objective of the research protocol reported here is the modelling and planning of interventions which are hypothetically feasible and effective for healthy lifestyle promotion in PHC, following four fundamental principles: (1) cooperation between primary care professionals, community partners and researchers, from the design stage; (2) reorganisation of the PCCs in order to facilitate the incorporation of health promotion; (3) adoption of a socio-ecological model, in which the health service plays an important role, complementary to that of other sectors and non-health resources; and (4) use of an appropriate methodological framework for the design and evaluation of complex interventions [1].

## Methods

### Objectives

1) To design innovative programmes for promoting at least two healthy lifestyle behaviours (physical activity, balanced diet, giving up smoking or the moderate consumption of alcoholic drinks), which are hypothetically effective, feasible and sustainable in routine PHC.

2) To evaluate the changes associated with the planning process in the actual healthy lifestyle promotion practice of PHC professionals, attitudes towards prevention and health promotion, and factors associated with the optimisation of such clinical practice.

### Design

A phase I formative research [43] will be performed in four PHC centres of the Basque Health Service – OSAKIDETZA. In a first descriptive stage, a strategic needs assessment will be carried out along with the prioritisation of the areas of preventive practice to be optimised and selection of the aims of the programmes. In a second, creative stage, specific objectives of intervention will be identified based on theoretical models of behaviour change and collaborative design of specific intervention actions for addressing and managing multiple risk behaviours will be undertaken.

Within the formative research, a quasi-experimental pre-post evaluation study will be performed to compare the changes in indicators related to health promotion practice in the four PCCs assigned to the aforementioned formative research process (Intervention Group) and those of other four centres, matched in structural and population characteristics (Control group) [46]. Additionally, Nominal Groups will be performed with professionals belonging to intervention centres, in order to identify the successes, strengths, weaknesses and shortcomings of the process, as well as experiences concerning its usefulness, and difficulties and barriers with respect to the cooperation between researchers and clinicians and between professions within the centres.

The project was approved by the Institutional Primary Care Research Committee of the Basque Health Service/Osakidetza, on 12^th ^April 2007, by the Basque Country Clinical Research Ethics Committee (Ref: 06/2008), and evaluated and approved for funding by the Basque Government Department of Health (Ref: 2007111009).

### Participants

#### Eligibility criteria

The particular focus of this study, which aims to introduce a process of change in health promotion practice in PHC under a framework of participative research with health professionals, community partners and researchers, determines the two characteristics that define the participants: (1) the intervention unit is the PCC; and (2) the centre must be especially interested in health promotion. The creative work to be undertaken requires that the professionals and health centres be positively motivated with respect to changing risk behaviours of their patients and the integration of health promotion in daily practice. That is why the following factors were considered in order to identify the centres that were eligible: (1) previous history of participation or evaluation of programmes for health promotion in the last 10 years, (2) previous initiatives of preventive practice optimisation and the promotion of healthy lifestyles, (3) existence of programmes, resources, services or initiatives for health promotion in the community or geographical area of reference, (4) positive attitude to working together and cooperation between the professionals of the centre, (5) centres in which it would be possible to make some kinds of changes in the organisation of services, once the potential innovative strategies for approaching multiple risk behaviours have been determined. The commitment of cooperation by a majority plus one of all the professionals of the centre for each of the professions (administrative officers, nurses, family doctors, paediatricians and others) was required to participate in the project.

#### Recruitment process of the Health Centres and PHC professionals

**Figure 1:**
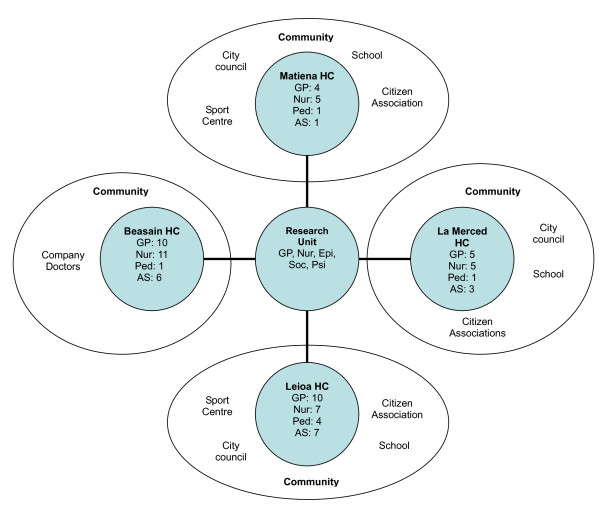
**Operational organization of the formative process "Prescribe Vida Saludable" in the intervention centres**. **Note**: HC, Health Centre; GP, General Practitioner; Nur, Nurse; Ped, Paediatrician; AS, Administrative Staff; Epi, Epidemiologist; Soc, Sociologist; Psi, Psychologist. Participation in Control health centres, Beraun HC: GP (5), Nur (4), Ped (1), AS (2), Social worker (1); Markina HC: GP (5), Nur (5), AS (3); Berango HC: GP (2), Nur (2), Ped (1), AS (1); and Zurbaran HC: GP (5), Nur (8), AS (3).

The identification of the eligible centres was performed via the Medical Directors of the seven divisions of primary care services, under which the PHC of the Basque Health Service – OSAKIDETZA is organised. Four of them accepted to collaborate and they each selected two primary care centres with similar structural and population characteristics. Project presentation sessions were given to the professionals of the selected centres, in which members of the research group explained the scientific and methodological details of the project, and the work plan. Then, each of the professionals were invited to collaborate through the presentation of an informed consent form. The majority of the professionals of each profession agreed to collaborate in eight of the centres; four of them being assigned for convenience to the formative research process, and the other four forming the reference or Control Group. Finally, 130 professionals (82 in the Intervention Group and 48 in the Control Group), collaborated in the project: 46 family doctors (29 in the Intervention and 17 in the Control), 45 nurses (26 in the Intervention and 19 in the Control), 27 administrative officers (18 in the Intervention and 9 in the Control), 10 paediatricians (8 in the Intervention and 2 in the Control), 3 midwives (2 in the Intervention and 1 in the Control group) and 1 social worker (Fig 1).

#### Recruitment process of community partners

The professionals belonging to centres assigned to the realisation of the formative process (Intervention Group), were asked to identify partners and resources in their community related to the promotion of healthy lifestyles. An initial list of resources and community partners for each health centre was drawn up. The members of the research team then contacted them by letter in which they were informed of the project and invited to collaborate by attending the activities within the formative process. A total of 30 community resources or agents were contacted, of which finally 18 agreed to collaborate, belonging to following fields: company doctors (2), citizen associations (4), professionals from the educational sphere (4), from municipal organisations (6) and from sports centres or facilities (2). (Figure 1).

### Formative Research

It will entail the realisation of 12 sessions directed by external facilitators and fed with selected information (reviews of scientific evidence, selected published literature, etc.) depending on the objective of each session [see Additional File 1]. These sessions respond to different strategies for the optimisation of clinical practice: needs assessment based on audit and feedback, prioritisation of areas of improvement using consensus techniques, evidence-based education sessions, and participatory modelling of interventions, by mapping and piloting of intervention components. The sessions will have a duration of approximately 90 minutes each and will be performed at intervals of at least 3 weeks, over the course of 12 months. In each centre, a coordinator will be identified to perform the necessary organisational tasks and they will be person responsible for communication with the research team. Before each session, they will be given and will distribute to the rest of professionals a summary of the previous session and the objectives of the present one, as well as selected documentation and support materials. After the execution of the sessions, synthesis documents summarising the process undertaken, the content of the discussions, and the results achieved, will be produced. In order to guarantee the accuracy of summaries, a circulation system of these synthesis documents between collaborating professionals will be implemented, to allow verification of the validity or to effect clarifications and changes in the content, results and conclusions obtained. The 12 sessions will be organised in two stages: a descriptive one (the first 4) and a creative one (the remaining 8) [see Additional File 1].

#### Descriptive Stage

In order to prioritise areas for optimisation in healthy lifestyle promotion taking into account the real-world context of the participating health centres, a process of strategic needs assessment will be carried out followed by a process for reaching consensus. The needs assessment in each centre will be based on two sources of information. On the one hand, a transversal descriptive study of the actual practice, skills, knowledge, perceived barriers and resources in relation to healthy lifestyle promotion by health centre professionals. On the other hand, data analysis will be performed on the prevalence of risk behaviours in the population and reception of preventive practice as reported by the users at the level of the health region of each collaborating centre, the information being taken from the Health Surveys of the Basque Autonomous Region in 2002 and 2007 [47,48]. The results of the needs assessment will be presented and discussed with the professionals and the community partners of each centre to gather their opinions concerning the presented indicators, the strengths, weaknesses, opportunities and threats for the centre and community, and possible areas for optimisation.

Subsequently a process for reaching consensus will be performed to identify and prioritise areas for optimisation in healthy lifestyle promotion focused on at least two risk behaviours with high prevalence or high priority in a specific target population. For this, the Nominal Group technique [49] will be used in which, after the identification of proposals by each of the professionals of the centre and community partners involved, the prioritised selection of one area of optimisation will be undertaken through two rounds of evaluation of the proposals on the basis of four dimensions: the magnitude of the problem, significance or potential impact, vulnerability and feasibility, following the method proposed by Hanlon [50]. The area prioritised for optimisation in each centre will be the goal for healthy lifestyle promotion, the object of the creative stage for the design and modelling of feasible and sustainable interventions.

#### Creative stage

In a second stage, we will proceed to the design and participative modelling of innovative interventions for addressing and managing multiple risk behaviours that could feasibly be implemented in a sustainable way in the real context of PHC. For this, two communication sessions on the evidence relating to risk behaviour modification will be organised, supported by selected documentation derived from the reviews of the scientific literature undertaken in the preclinical phase [51]. The sessions will have the following content: a) theoretical models of healthy lifestyle modification; and b) evidence of effective intervention strategies, in which effective models and strategies for addressing and managing risk behaviours in PHC will be described.

On the basis of the theoretical models and intervention strategies described in the aforementioned sessions, professionals of the centre will perform an analysis prior to the programme planning to identify the potential specific objectives – personal, interpersonal and environmental factors that should be worked on to begin and maintain the process of behavioural and environmental change – as well as potential concrete intervention actions – strategies or measures intended to modify these aforementioned specific objectives or key factors [16-18].

The proposal presented by the professionals concerning the specific intervention objectives and strategies will be analysed, reworked, and completed by the members of the research team. Afterwards, with the objective of concluding the prior analysis of the programme, the professionals of the centre and community partners will proceed to the determination of potential strategies for translating and sustainably introducing the plan to the real-world conditions of the centre and the community. For this, the professionals of the centre and community partners potentially involved in each intervention action will be identified, as well as the necessary resources and the sequence of implementation of intervention: WHICH action is to be undertaken by WHOM, HOW, WHEN and WHERE. To facilitate this exercise, the members of the research team will produce selected documentation concerning action-research initiatives at an international level related to programmes for addressing risk behaviours in a clinical context, with the objective of serving as an example or suggesting new ways of translating research into practice, and of introducing and coordinating intervention strategies and actions.

The essential active components of the preliminary programme for healthy lifestyles promotion will be progressively piloted in the real-world conditions of the health centres for a period of between one and three months. For this, the professionals of the centre will select intervention objectives and actions to pilot on the basis of their importance in the process of behavioural change, their innovative nature or uncertainty over their true feasibility. Then, pilot loops along with their associated evaluation sessions will be undertaken. Subsequently, a structured group discussion session will be organised to analyse the real feasibility of the strategies and actions piloted, in order to optimise their potential impact and sustainable integration. With respect to these intervention strategies and actions, the key components and processes will be identified. Barriers and resources in relation to the context of each centre (personal, material or organisational) that make it easy or difficult to set the process of implementation in motion, and necessary changes and readjustments will also be identified.

On the basis of the feasibility evaluation regarding intervention actions and implementation strategies, and from the overall conclusions of the formative process undertaken in each centre, a final selection and standardisation of the following intervention programme components will be implemented: a) operational objectives of the programme; b) target population for the intervention (criteria for eligibility and exclusion) and mechanisms for addressing them; c) personal factors and characteristics (socio-demographic, biological, health status, risk status, lifestyle, beliefs and attitudes, family, etc.) essential to evaluate in order to adapt and guide the process and content of the intervention; d) key components of the intervention: strategies or processes; personnel involved; material or organisational resources needed, with respect to the context of each centre; combination of these components; and intensity of the intervention.

### Comparison Groups

The professionals of the participating centres who have been included in the Control Group will maintain the usual standard care for healthy lifestyle promotion in the Basque Health Service – OSAKIDETZA and will not be the object of any formative process or intervention.

### Evaluation

The primary outcome measure for the evaluation of the impact of the formative process will be the professionals' self-reported healthy lifestyle promotion practice, assessed through an updated version of the *Preventive Activities Questionnaire *[52]. This same instrument will be used to assess the following factors associated to healthy lifestyles promotion: attitude towards the promotion of healthy lifestyles, perceived barriers, knowledge, skills and confidence for addressing healthy lifestyles. Additionally, the organizational climate in the health centre will be assessed using a translated and culturally adapted version of the *Survey of Organizational Attributes for Primary Care (SOAPC) *[53].

The *Preventive Activities Questionnaire *[52] was originally developed in the framework of a research project whose objective was to estimate how frequently preventive activities recommended by the main consensus groups were put into practice in the PCCs of the Autonomous Region of the Basque Country. In order to update the questionnaire, a panel of five experts in health promotion was assembled from PHC professionals (family doctors and nursing personnel), epidemiologists, psychologists and sociologists. The expert panel revised the original questionnaire and suggested changes and new questions on the basis of: 1) previous experience in application of the tool, for which the authors of the questionnaire were interviewed; 2) the results of the previous formative research (preclinical phase) regarding the integration of healthy lifestyle promotion in PHC [1]; and 3) a rapid review of tools for assessing preventive practices in the context of PHC. The research team drew up a first version of the questionnaire which was then submitted to the expert panel. They were asked to evaluate: the content validity, that is, whether the items within the questionnaire were capable of evaluating all the dimensions intended to be measured; the logical order of the questions and the sections; their grammatical quality; and the adequacy of the scales for each item punctuation. The questionnaire was reedited according to the contributions given by the expert panel.

For the evaluation of the centre as an organisation and, in particular, the perception of the professionals of the organisational climate, a translated and culturally adapted version of the *SOAPC *[53] will be used. A structured process of translation and back-translation of the original version of the questionnaire was performed by two bilingual researchers independently. This process yielded two translated versions that were quantitatively evaluated by each of the members of the expert panel independently. Each member had to judge the two versions on whether concepts expressed were equivalent, their clarity and the naturalness of the style of the writing. Subsequently a consensus meeting was used to reach agreement on a unique new version of the questionnaire, which was subsequently discussed with the authors of the original questionnaire.

A piloting of the two questionnaires was undertaken with 21 professionals of the most representative professions of the health centre (4 administrative officers, 10 nurses and 7 doctors). After the pilot trial, to obtain the final version, the questionnaire was again submitted to evaluation by the expert panel, and after the analysis, some questions were rewritten and some others removed, for not returning relevant information.

The updated version of the *Preventive Activities Questionnaire *(UIAPB) is divided into 7 sections. It consists of 120 items and investigates the following dimensions: 1) attitude towards healthy lifestyle promotion in PHC; 2) current self-reported practice of healthy lifestyle promotion; 3) knowledge, skills and perceived effectiveness in addressing risk behaviours; 4) resources for healthy lifestyle promotion in the consulting room/PCC; and 5) perceived barriers to health promotion. The translated and culturally adapted version of the SOAPC comprises 19 items which measure 5 relevant organisational factors: 1) communication: ability of the professional to work in a team when problems and differences arise; 2) practice-wide decision-making: participation of the professionals of the centre in decision-making; 3) nurses' participation in decision making: participation specifically of the nursing staff in decision-making; 4) stress/chaos: workload of the professionals of the centre; and 5) history of change: history of organisational changes and/or culture within the centre.

The indicators related to the formative programme planning process to be evaluated are based on the RE-AIM model: a) Reach: percentage of participating centres, professionals and partners (among the eligible ones), and their representativeness; b) Efficacy and evaluation of the experience: impact of the formative process on healthy lifestyle promotion practice of the participating professionals and their perception concerning the usefulness of the formative process and its results; c) Adoption and implementation: degree of execution of the components of the formative process as planned: number and duration of the sessions, number of participants in each session, etc.; d) Maintenance and monitoring: degree of continuation of the formative process components and changes made; percentage of participants leaving the study, their representativeness and impact on the Reach, Adoption and Efficacy of the formative process [54].

### Analysis

In order to evaluate the changes associated with the formative process, quantitative comparisons will be performed between the professionals assigned to the Intervention Group and those from the Control Group, on an intention to treat basis. The means of continuous variables will be compared using the Student's t test and the proportions using the Chi-square test. The differences between the two groups will be estimated and the 95% confidence interval calculated. The comparisons will be adjusted for potential confounding factors by stratified analysis. Finally, multivariate statistical models, linear for continuous variables and logistic for proportions, will be constructed in order to simultaneously account for possible confounding effects, following a "forward strategy" guided by the aforementioned stratified analysis. The sample size of the present formative research study (130 health professionals, 30% in the control group) will provide a greater than 80% power to detect as significant (alpha = 0.05) a difference of at least 20% (improvements of 5% and 25% in the control and intervention groups, respectively) in the proportion of professionals that report to ask and advice about healthy lifestyles to the mayority of their patients.

## Discussion

This project tackles one of the most difficult challenges for the primary care services: changing the practice of heath promotion. Scientific literature about the change and the optimisation of clinical practice agrees on highlighting four factors that are important and necessary in achieving this change: (1) the commitment of the professionals and partners involved; (2) their competence for change, such as new knowledge and analytical skills; (3) the active and participative cooperation of the interdisciplinary partners involved throughout the process of change; and (4) the necessary resources to facilitate this change [55-58].

The first two factors, commitment and competence, are essential but not sufficient. The descriptive stage of the present project as well as sessions 5 to 7 of the creative stage, are directed towards those two factors. It includes the reflection induced by the study and discussion sessions of the potential impact of healthy lifestyle promotion compared to the evaluation of current practice, the prioritisation of needs, the selection of a common aim for the whole centre, dissemination of the evidence-based knowledge, etc. In addition, the specific context, with respect to the resources available and potential barriers for each centre in particular and for the health system in general, is being taken into account, as recommended by the experts.

We think that the most serious threats to the current project are failing to maintain the active cooperation of the professionals and partners, in an environment characterised by work overload, and the rigidity of the system of provision of health care, with continuous change of personnel and an absence of incentives beyond personal motivation. We expect that these problems will arise in the planning and pilot stage of sessions 8 to 12. In order to address this huge challenge, a great effort has been made to obtain the support of people and key partners within the system (managers and directors) and outside the system (community). An initial selection of the centres especially interested has been performed and we are going to use incentives such as formative accreditation (continuous professional development). However, in order that the promotion of health is adopted in a successful way in the daily practice of PHC via action research initiatives such as this one, we believe that it will be necessary to count on additional incentives such as professional acknowledgement, scientific rewards or financial incentives.

## Competing interests

The authors declare that they have no competing interests.

## Authors' contributions

GG and AS conceived the idea and are the study guarantors. They are primarily responsible for the study design and planning, obtained funding, will be responsible of project coordination and supervision, analysis and interpretation of results and manuscript preparation. JMC and HP collaborated in the study design, obtained funding, and will be responsible for study coordination, interpretation of results and manuscript preparation. LB and CC will be responsible for the analysis of results of the discussion groups and critically reviewed the manuscript. All contributors approved this version submitted for publication to the BMC Health Services Research. 

## Pre-publication history

The pre-publication history for this paper can be accessed here:



## Supplementary Material

Additional file 1 **Table S1 – Operational design of the formative process for intervention modelling and planning**. Structured representation of the sessions that compose the "Prescribe Vida Saludable" formative process. Click here for file
