# Supplementary material for: Modelling innovative interventions for optimising healthy lifestyle promotion in primary health care: "Prescribe Vida Saludable" phase I research protocol
Source: BMC Health Serv Res. 2009 Jun 18;9:103. doi: 10.1186/1472-6963-9-103 (PMC2714033; doi:10.1186/1472-6963-9-103)
Supplement: Additional file 1 — Table S1 – Operational design of the formative process for intervention modelling and planning. Structured representation of the sessions that compose the "Prescribe Vida Saludable" formative process. [file 1472-6963-9-103-S1.doc]

**Table 1. Operational design of the formative process for intervention modelling and planning**

| **PROCESS** | **PREVIOUS DOCUMENTATION** | **RESULTS** |
| --- | --- | --- |
| ***DESCRIPTIVE STAGE*** |
| *Session 1: Presentation of the “Prescribe Vida Saludable” project (PVS): the problem of health promotion in primary care*  Objective: To present the research project to the professionals of the centre, describing its theoretical justification, methodological and operational contents, in order to achieve their collaboration  Method: Oral presentation with interactive media and documentation | Study protocol and operating manual  Prescribe Vida Saludable preclinical phase manuscript [1] | Informed consent to participate of at least half of the professionals of each profession strata in each centre |
| *Session 2: Measurement and description of needs: current healthy lifestyle promotion in the centre*  Objective: To describe the magnitude and relevance of the problem of health promotion in the collaborating centre, in terms of gap between the current and the optimum state:  a) attitude, knowledge and real practice with respect to healthy lifestyle promotion  b) health status and prevalence of risk behaviours in the users of centre and local population  Methods: Oral presentation of the needs assessment using interactive media and documentation followed by group discussion | Baseline measurement of: healthy lifestyle promotion practice, attitude, skills, knowledge, barriers and organisational climate  Report of the prevalence of risk behaviours in the population and preventive practice stated by users at the health region level [47,48] | Strategic needs assessment and perception of the professionals concerning indicators, potential areas for improvement, strengths, weaknesses, opportunities and threats |
| *Sessions 3 and 4: Identification and selection of areas prioritised for improvement*  Objective: To prioritise an area of improvement for healthy lifestyle promotion within the centre, directed towards at least two risk behaviours in a specific target population.  Methods: Structured session for identification and prioritisation of proposals based on the Nominal Group technique | Practice guidelines for healthy lifestyle promotion in primary care  Methodology and criteria for the identification and selection of areas for optimisation | Commitment with an aim for healthy lifestyle promotion, arrived at by consensus |
| ***CREATIVE STAGE*** |  |  |
| *Sessions 5-7: Theoretical models of healthy lifestyle promotion, effective intervention strategies and integration methods*  Objective: To describe theoretical knowledge and available scientific evidence concerning effective strategies and factors in the change of risk behaviours  Methods: Evidence-based training sessions followed by group discussion. Analysis exercise prior to the planning of strategies for intervention. | OSTEBA Preclinical phase Report of the PVS project [51]  Selected documentation reviewing evidence concerning effective intervention strategies for healthy lifestyle promotion  Selected scientific literature concerning translation of strategies to clinical practice | Analysis prior to the planning of intervention strategies: identification of specific objectives, actions, agents and resources involved |
| *Sessions 8 to 11: Selection, articulation and piloting of intervention actions*  Objective: Identification, description and selection of intervention actions and strategies for addressing and managing risk behaviours, on the basis of their piloted feasibility and viability.  Methods: Structured discussion sessions. Practical exercise for the identification of feasibly implementable strategies. Progressive piloting loops of intervention actions and implementation strategies in real-world conditions, with their associated monitoring sessions | Protocols for pilot trial of intervention actions: objectives of the piloting; measures and processes; trial planning and evaluation | Report of the intervention strategies piloting process and proposals for improvement |
| *Session 12: Final selection of intervention strategies and healthy lifestyle promotion programme planning*  Objectives: Standardisation of a preliminary programme for healthy lifestyle promotion on the basis of the feasibility evaluation of its essential components and the resources necessary for its implementation  Methods: Structured consensus evaluation session based on the Nominal Group technique | Report of the feasibility of piloted intervention components and implementation strategies | Potentially effective intervention programme for healthy lifestyle promotion in PHC |
